# Prehistoric agriculture and social structure in the southwestern Tarim Basin: multiproxy analyses at Wupaer

**DOI:** 10.1038/s41598-020-70515-y

**Published:** 2020-08-28

**Authors:** Qingjiang Yang, Xinying Zhou, Robert Nicholas Spengler, Keliang Zhao, Junchi Liu, Yige Bao, Peter Weiming Jia, Xiaoqiang Li

**Affiliations:** 1 Key Laboratory of Vertebrate Evolution and Human Origin, Institute of Vertebrate Paleontology and Paleoanthropology, Chinese Academy of Sciences, Beijing, 100044 China; 2 CAS Center for Excellence in Life and Paleoenvironment, Beijing, 100044 China; 3 University of Chinese Academy of Sciences, Beijing, 100049 China; 4 Max Planck Institute for the Science of Human History, 07745 Jena, Germany; 5 Department of Archaeology, The University of Sydney, Sydney, NSW 2006 Australia

**Keywords:** Palaeontology, Archaeology

## Abstract

The oasis villages of the Tarim Basin served as hubs along the ancient Silk Road, and they played an important role in facilitating communication between the imperial centers of Asia. These villages were supported by an irrigated form of cereal farming that was specifically adapted to these early oasis settlements. In this manuscript, we present the results from new archaeobotanical analyses, radiocarbon dating, and organic carbon isotopic studies directly from carbonized seeds at the Wupaer site (1500–400 BC) in the Kashgar Oasis of the western Tarim Basin. Our results showed that early farming in the oasis relied on a mixed wheat and barley system, but after 1200 BC was intensified through more elaborate irrigation, the introduction of more water-demanding legumes, and possibly a greater reliance on free-threshing wheat. These crops and the knowledge of irrigated farming likely dispersed into the Tarim Basin through the mountains from southern Central Asia. Improved agricultural productivity in the Tarim Basin may also have led to demographic and socio-political shifts and fed into the increased exchange that is colloquially referred to as the Silk Road.

## Introduction

**Figure 1:**
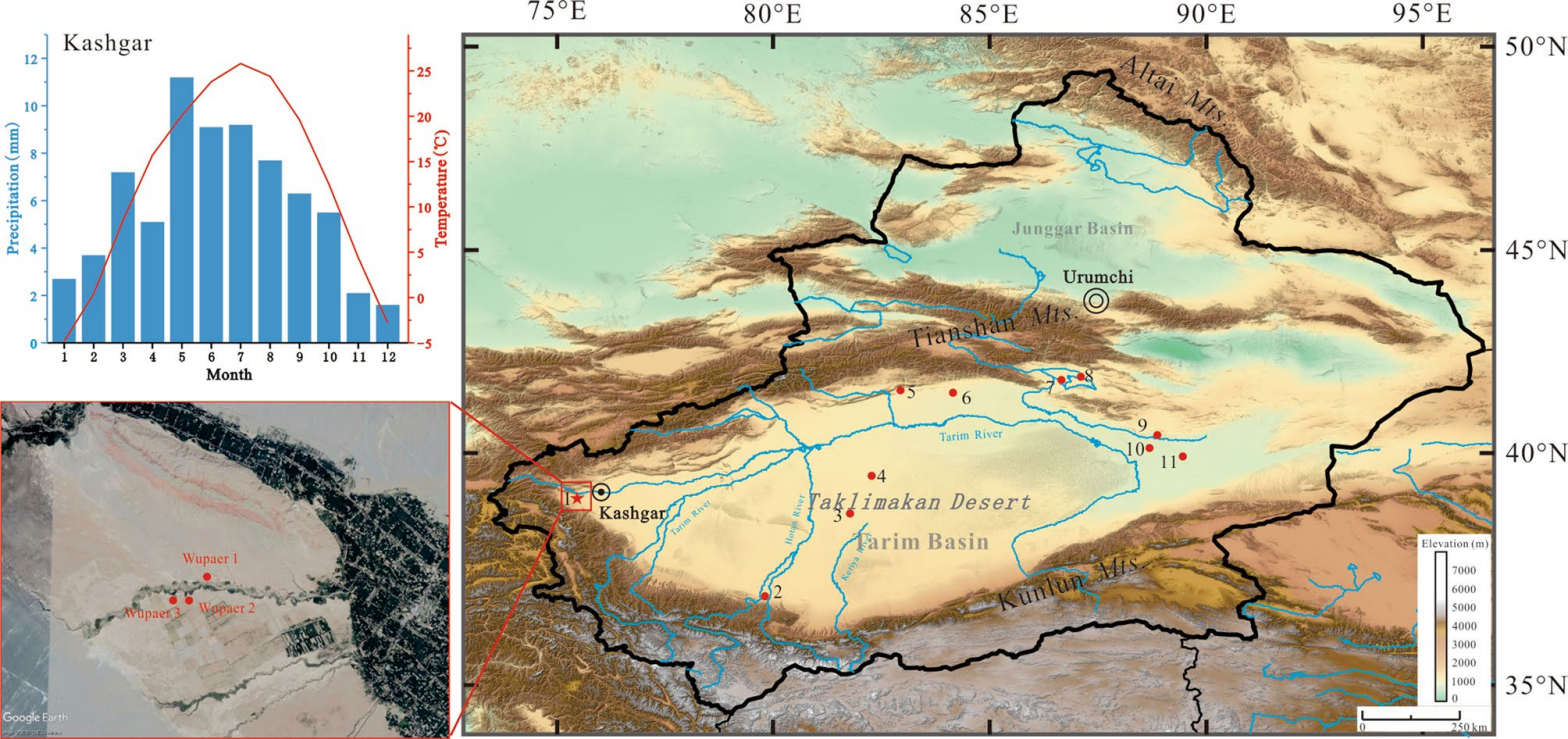
Archaeological sites with ancient remains of crops in Xinjiang (DEM date derives from Geospatial Data Cloud https://www.gscloud.cn and the DEM date is edited by Global mapper; the satellite image is from Google Earth). (1) Wupaer (this study), (2) Sampula Cemetery (late first millennium B C), (3) Yuansha ancient city (Djoumboulak Koum, ca. 400–0 BC), (4) North Keriya (close to the Xiaohe Cemetery), (5) Qiuci ancient city, (6) Qunbake Cemetery (955–680 BC), (7) Yanqi ancient city, (8) Xintala (1920–1530 BC), (9) Gumugou Cemetery (1886–1746 BC), (10) Xiaohe Cemetery (1691–1292 BC), and (11) Loulan ancient city (ca. 200 BC–400 AD).

The Tarim Basin is located at the central point of the historic Eurasia trade routes; resting in the rain shadows of the Kunlun and Tianshan mountains, it is one of the driest and least arable areas in Asia. The Taklimakan Desert occupies the middle of the basin, and while receiving almost no rainfall, it evaporates up to 3,000 mm per annum. Glacial-melt streams from the mountains flow into the desert and mostly disappear in its hinterland. This landscape gave birth to the beaded oases that characterized the historic Silk Road. Archaeological and historical evidence attests to the presence of agricultural city-states or politically organized villages in these oases, likely being founded as early as the fifth century BC^1^. Archaeologists have suggested that many of these urbanized oases housed populations of thousands to tens of thousands, with some notable examples including Qiuci, Yumi, Yanqi, and Loulan (Fig. 1)^2,3^. Historians refer to these pockets of dense human occupation as the “Western Regions”^2,3^. Prehistorically, these oases played a prominent role in cultural exchange and diffusion during the early globalization process^4–10^. These city-states helped connect the two ends of ancient Eurasia and were the main channel between peoples in the regions of northern China and southwest Asia^11–14^. For decades, archaeologists have recognized cultural similarities between ancient peoples in the Tien Shan Mountains, Pamirs Plateau, Ferghana Basin, and those that lived across the oases of Xinjiang^12,15,16^. The study of the origin and evolution of these city-states, their cultural adaptations, and their response to climate change has been the focus of global historians, archaeologists, and paleoclimate scholars^9,17^.

Since the Swedish archaeologists, Bergman, first found the well-preserved mummies of the Xiaohe Cemetery in Lop Nur in 1934^18^, new prehistoric settlements and tombs have continually been discovered, including the Gumugou Cemetery^19,20^, Xintala^21,22^, Yuansha^23–26^, North Keriya Cemetery^27^, and Yanghai^28,29^ (Fig. 1). Through extensive excavation, archaeologists have gradually revealed culture aspects of the prehistoric people of the Tarim Basin. It is now clear that they existed in small groups of agricultural or semi-agricultural peoples concentrated in oases by as early as 2000 BC^21,22^. After 500 BC, these geographically confined populations expanded into prosperous city-states^23–26,30^ (Fig. 1), which consisted of one town (often walled), the immediate agricultural vicinity, and a stratified population^17^. After roughly a millennium and a half of human occupation in these oases, farming communities developed grain production and herding strategies intensive enough to support the demographic upturns seen in the mid-first millennium BC. Most scholars believe that exchange along more organized trade routes of the Silk Road began around this time^5,31^, and commercial expansion could also have fueled greater political organization and population growth in these oases. In this article we present new data to better understand the changes in social orders that marked the late first millennium BC transition in the Tarim Basin.

New archaeobotanical research in northwestern China is illustrating how complex ancient farming strategies were, with different groups maintaining their own crop repertoires and regionally stable preferences in grain choices over long periods of time^7,31^. The magnitude of archaeological, especially archaeobotanical, research in Xinjiang has increased over the past couple decades^20,32,33^. However, most of this scholarly attention has focused on the eastern or northern portions of Xinjiang^10,34–36^ (Fig. 1). Due largely to the difficulty in accessing southern Xinjiang, the development of social systems and agricultural practices in this region, especially to the southwest of the Tarim Basin, remains largely unexplored. In this study, we present new archaeobotanical data, combined with carbon isotope evidence directly from ancient grains and novel radiocarbon dates from the ruins of the oasis town of Wupaer in the Kashgar Oasis of the western Tarim Basin. These datasets inform important new perspectives on long-standing debates relating to political transitions and demographic shifts at the Bronze/Iron Age Transition in Central Asia.

The Kashgar Oasis is located along the western margin of the Tarim Basin (Fig. 1). The Oasis is characterized by an extreme intercontinental climate and hyper aridity, with a mean annual temperature of 11.8 °C, where the mean temperatures in the coldest month (January) and hottest month (July) are 4.8 °C and 25.8 °C, respectively. The annual total precipitation is 71.4 mm, but annual total evaporation is estimated at close to 3,000 mm^37^ (Fig. 1). The Yarkant River originates in the Kunlun Maintains and flows throw the Kashgar Oasis, ultimately emptying into the Tarim River (Fig. 1). Two major arteries of the ancient Silk Road of the Western Han Dynasty (202 BC–8 AD) joined at Kashgar, tracing the northern and southern margins of the Taklimakan Desert and then traversed the Pamir Plateau into Central Asia^38^.

The cluster of archaeological sites that make up Wupaer (39°18′42.11″N, 75°27′45.41″E), also known as Aketala, Wenguluoke, Keluketala, and Dewaleke, has an area of 10,000 m^2^, and is located 5 km west of the modern town of Wupaer and 50 km southwest of Kashgar (Fig. 1). The ancient settlement was first explored archaeologically in 1972 and reported in publication in 1977. A large number of stone artefacts and pottery fragments were exposed on the surface of the site. The stone artefacts included stone knives, hammer axes, sickles, millstones, pestle, balls (likely sling balls), and arrowheads; the ceramics were dominated by a sand-pottery type^1,39^. Due to a poor understanding of ceramic chronologies for Xinjiang, these sites were first reported as Neolithic, but the Aketaka city ruins, surrounding the Wupaer site, are now considered to be an early historic site^39^. Therefore, the cultural properties, chronology, and duration of occupation for the site have not been formally determined, but they clearly represent a palimpsest of wind-deflated cultural layers and reworked sediments.

## Results

### Chronology

The results of radiocarbon dating are showed in Table 1; the seven age ranges span from 1508 to 418 cal BC, but there appears to be a gap in occupation between ca. 1300–1200 cal BC, which may be a result of the contexts available for sampling or represent a period of less intensive occupation. The seven dates allow us to discuss the rough chronology of archaeobotanical remains that we present here and group them into the early Wupaer period (1500–1300 BC) and the late Wupaer period (1200–400 BC).

**Table 1:** The results of AMS^14^C dating of the Wupaer site.

Site	Lab no	δ^13^C (‰)	Material	Age (year BP)	Calibrated age (BC)
Wupaer 1	OZK661	− 23.0 ± 0.1	Wheat	2,540 ± 50	808–427
Wupaer 1	OZK660	− 23.1 ± 0.1	Wheat	2,595 ± 50	892–542
Wupaer 2	OZL443	− 23.4 ± 0.4	Wheat	2,497 ± 36	788–418
Wupaer 2	OZL442	− 24.5 ± 0.1	Wheat	2,856 ± 40	1,189–910
Wupaer 2	Beta-526294	− 24.4	Wheat	2,470 ± 30	768–476
Wupaer 3	OZL441	− 23.3 ± 0.1	Wheat	3,155 ± 39	1508–1,318
Wupaer 3	Beta-526293	− 23.9	Naked barley	3,160 ± 30	1501–1,390

### Carbonized seeds

**Figure 2:**
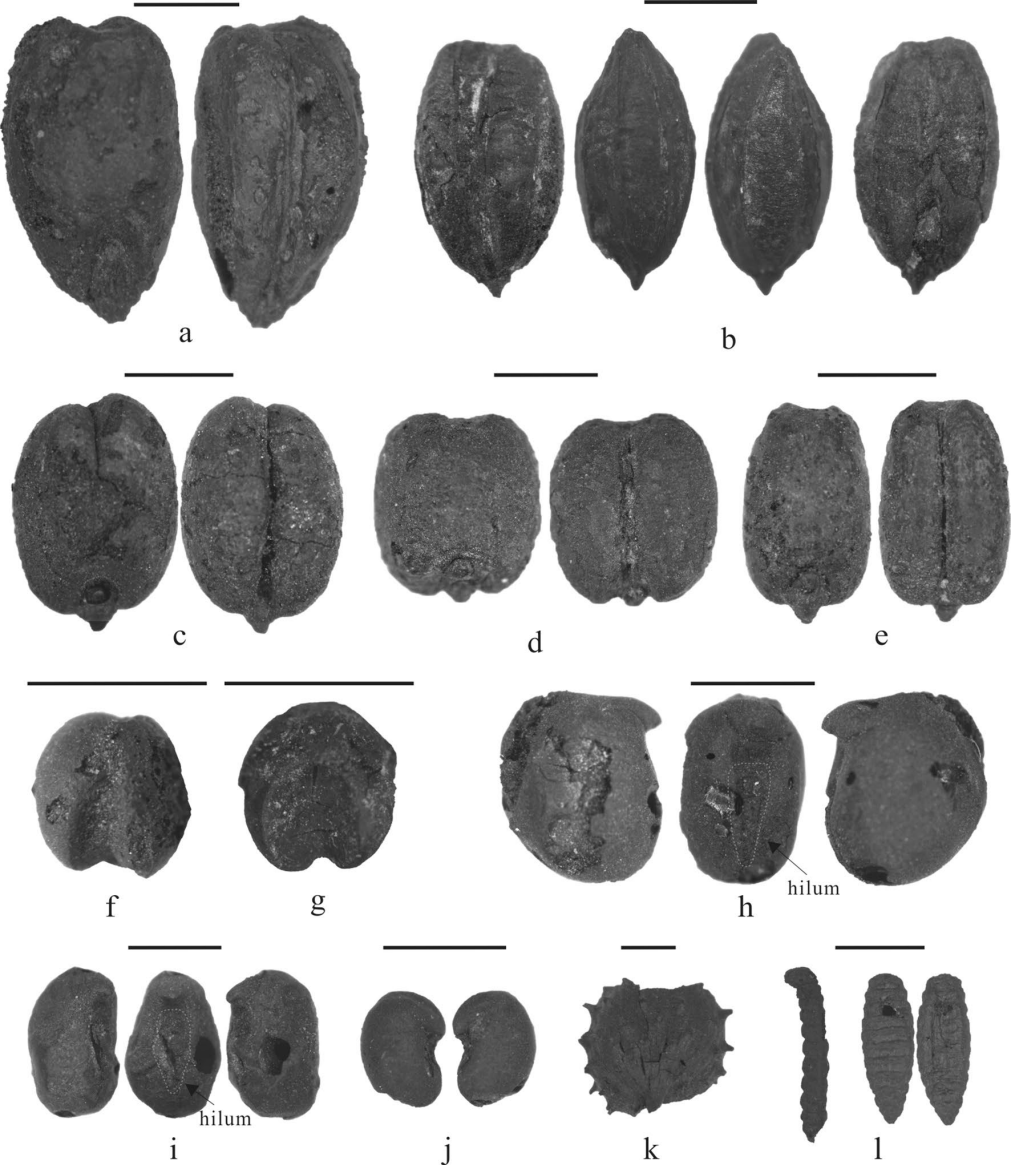
Carbonized remains from the Wupaer site. (**a**) two-rowed barley (Wupaer 2-1, 1189–418 BC), (**b**) six-rowed barley (Wupaer 2-3, 1189–418 BC), (**c**) naked barley (Wupaer 3-1, 1508–1318 BC), (**d**) highly compact wheat (Wupaer 2-3, 1189–418 BC), (**e**) common wheat (Wupaer 2-3, 1189–418 BC), (**f**) foxtail millet (Wupaer 1-1-2, 892–427 BC), (**g**) common millet (Wupaer 1-1-2, 892–427 BC), (**h**) pea (Wupaer 2-1, 1189–418 BC), (**i**) *Vigna* sp. (Wupaer 2-1, 1189–418 BC), (**j**) camel thorn (Wupaer 2-3, 1189–418 BC), (**k**) cockleburs (Wupaer 2-1, 1189–418 BC), (**l**) larva and pupa of lesser grain borer (Wupaer 2-3, 1189–418 BC). Scale bar is 2 mm.

A total of 643 charred seeds were identified from the Wupaer site, which included cereal crops, legumes, and wild herbaceous plants (Fig. 2 and Table S1). The carbonized crops consisted of barley (Fig. 2a–c), wheat (Fig. 2d,e), foxtail (*Setaria italica*) (Fig. 2f), and broomcorn millet (*Panicum miliaceum*) (Fig. 2g). The carbonized legumes consisted of peas (*Pisum sativum*) (Fig. 2h) and *Vigna* sp. (Fig. 2i). Wild plants consisted of camelthorn seeds (*Alhagia* sp.) (Fig. 2j) and cocklebur fruit casings (*Xanthium* sp.) (Fig. 2k). Two carbonized insect larvae were also recovered, which have tentatively been identified as grain borers, morphologically resembling lesser grain borers (*Rhyzopertha dominica*) (Fig. 2l).

### Barley (*Hordeum vulgare*)

Three types of barley were identified at Wupaer. Based on grain apex morphology, we believe that both two-rowed (*Hordeum vulgare* var. *distichon*) (Fig. 2a) and six-rowed varieties of hulled barley (*Hordeum vulgare* var. *vulgare*) (Fig. 2b) were present. Additionally, smoother grained and more compact specimens of naked barley (*Hordeum vulgare* var. *nudum*) (Fig. 2c) were present. All three types contained a fusiform morphology. The grains of two-rowed hulled barley were the largest of the three types, with grain sizes reaching 6.0, 3.3, and 2.7 mm in length, width, and thickness, respectively. The lateral grains of six-rowed hulled barley were easy to recognize by their twisted form; this variety had grains smaller than the two-rowed type, and the average length, width, and thickness measurements were 4.22, 2.32, and 1.82 mm, respectively. Central grains on a spikelet of six-rowed barley were indistinguishable from grains of two-rowed barley, hence, we cannot discuss proportions of the two forms. Naked barley was slightly larger than six-rowed barley, and had an average grain size of 4.49 mm in length, 3.21 mm in width, and 2.26 mm in thickness.

### Wheat (*Triticum aestivum* or *T. aestivum*/*turgidum*)

**Figure 3:**
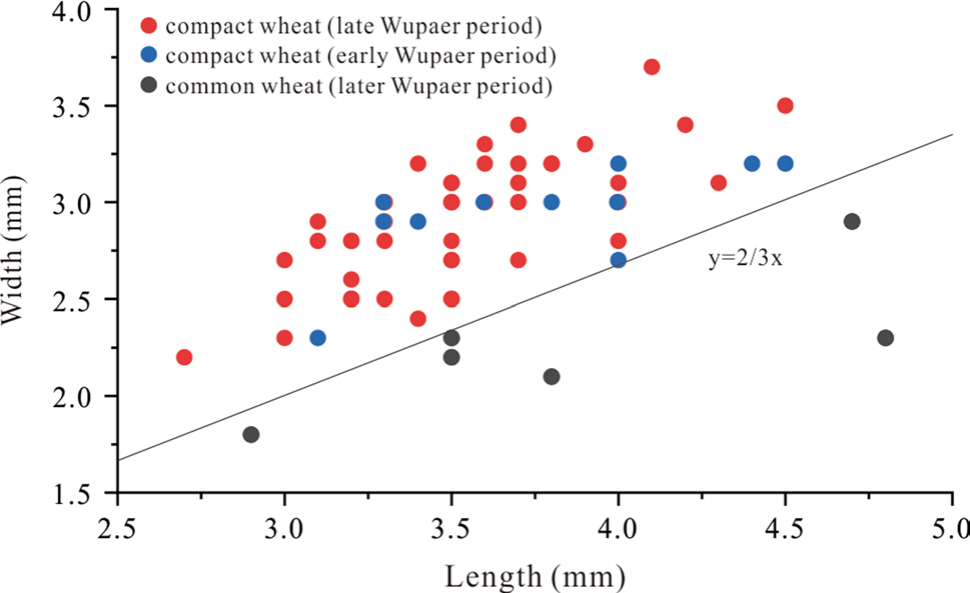
A scatter diagram of the length and width for compact and common wheat grains.

Wheat was the most abundant crop recovered from three of the pits, with a total of 548 carbonized grains in the assemblage, which accounted for 85% of the carbonized remains. The width/length ratio of most of the wheat grains was more than 2/3, suggesting that they fell into the identification index of compact morphotypes^40,41^ (Fig. 3). While landrace varieties of any crop express greater variability within a variety than modern crop varieties, the compact wheat grains from Wupaer (Fig. 2d) were nearly round and the sizes ranged from 2.4–4.5 mm in length, 1.6–3.7 mm in width, and 1.5–3 mm in thickness, the average width/length radio is 0.81. The handful of grains that we ascribed to the category of common wheat (Fig. 2e) displayed an oval shape and their size was 2.9–4.8 mm in length, 1.8–2.9 mm in width, and 1.5–2.0 mm in thickness, the average width/length radio was 0.58. Given the considerable variability between these grains, no clear break between the two populations, and the limited sample size, we cannot say with confidence if there were different morphotypes present or a high degree of variability within one form—possibly resulting from differences in watering regimes.

### Millets

Millet grains were not recovered from the earliest contexts from the site, which seems to imply that they were later introductions, possibly in the late second millennium BC. Millets would have been known in this region and are recovered from earlier contexts in the mountains of Central Asia, hence, additional studies are necessary to verify their absence in the early layers at the site. There were four foxtail millet grains (Fig. 2f) recovered from Wupaer. One of the grains was measured, with a size of 1.8 mm in length, 1.3 mm in width, and 1.2 mm in thickness. A total of 11 grains of broomcorn millet (Fig. 2g) were found at the Wupaer site. One of these grains was measured as having a size of 1.8 mm in length, 1.5 mm in width, and 1.0–1.1 mm in thickness.

### Wild and domesticated Fabaceae

There were 3 carbonized peas (Fig. 2h) found at Wupaer site, the average size of these legumes was 3 mm in length, 2.9 mm in width, and 2.0 mm in thickness. All of them had distinct radicals and embryo notches; they also had clear cotyledon splits. While we are cautious to make any interpretations based on two seeds alone, especially when we do not have representative comparative material for all wild Fabaceae in the region, there were two seeds that express some morphological similarity to cultivated *Vigna* sp. (Fig. 2i) as identified at sites in northern India and Pakistan^42,43^. Given the close proximity of Wupaer to the Kashmir Valley, where early *Vigna* have been identified, we think the possibility that there seeds represent a cultivated legume should be considered. Images of both seeds are presented in Fig. 2i, and they were both found at Wupaer 2-1, the size of seeds ranged from 3.1–3.2 mm in length, 2.2–2.3 mm in width, and 2–2.1 mm in thickness. Seven wild camel thorn (*Alhagi sparsifolia*) seeds were also recovered (Fig. 2j), with average sizes of 2.0–2.3 mm in length, 1.3–1.5 mm in width, and 1.1–1.2 mm in thickness. Camel thorn is commonly recovered in archaeobotanical assemblages across arid Central Asia and they express a wide range in morphological variability; hence, we can’t rule out the possibility that the two seeds mentioned above are exceptionally large *Alhagi* seeds.

### Organic carbon isotope rations in ancient grains

The δ^13^C values of 15 wheat seeds ranged from − 25.3 to − 21.8‰, and the average value and median were − 23.4‰ and − 23.6‰, respectively. The calculated Δ^13^C values of wheat seeds ranged from 15.8 to 19.4‰. The water input of wheat reconstructed by Δ^13^C values widely differ, from 71 to 215 mm, and average water input is 137 mm.

## Discussion

### Agricultural strategies between 1500–400 BC in the southwestern Tarim Basin

**Figure 4:**
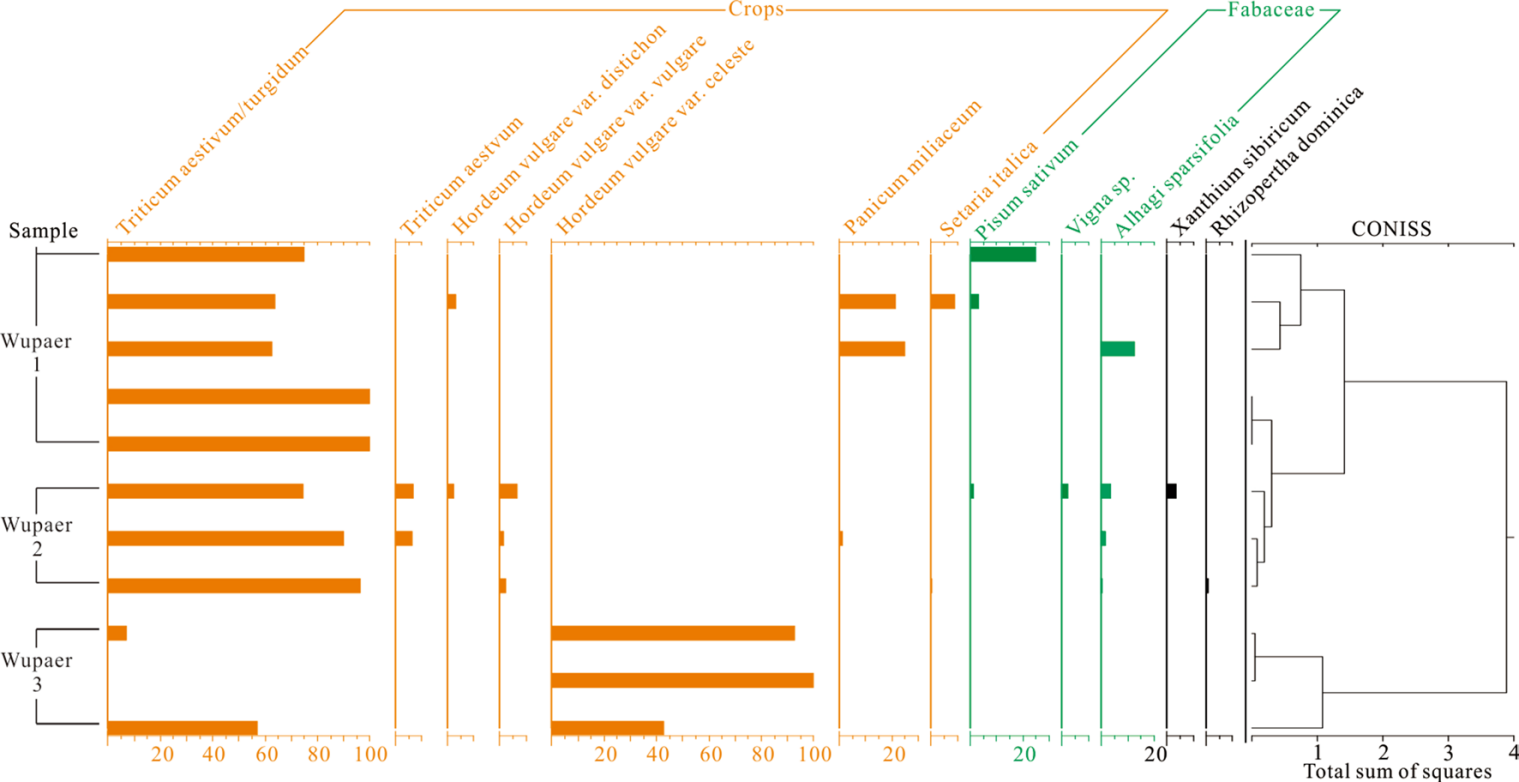
The spectrum of charred seeds from the Wupaer site.

The only crops recovered from sediments dating to the early phase of occupation at the Wupaer site (1500–1300 BC) were naked barley and compact wheat (Fig. 4). This is not surprising, given that increasing evidence has illustrated that these two grains were the earliest domesticated crops to spread through the mountains of Inner Asia^42^. The long-season cereals were likely well-adapted to environments of the more northerly latitudes and spread more rapidly eastward, than did legumes or other cereal varieties, such as tetraploid wheats. Both cereals spread into the Hexi Corridor, around the peripheries of the Tibetan Plateau, and eventually into the middle and lower reaches of the Yellow River by the end of the third millennium BC^44,45^. While we can only speculate about what the agricultural strategies in the Taklimakan Desert during this early period looked like, based on analogies with neighboring regions^44^, we believe that a low-investment form of agriculture, near river edges or spring promotes complimented simple herding strategies.

Supporting our results from early Wupaer, archaeobotanical data from Xintala (ca. 1900–1500 BC)^22^, also showed that the agriculture was dominated by compact free-threshing wheat and naked barley (Fig. 6a). At the Gumugou Cemetery (1886–1746 BC), wheat was the only crop recovered^20^, and the crop remains from the Xiaohe Cemetery^32^ and the microfossil analysis of a desiccated cake from the North Keriya Cemetery^25^ indicated that both wheat and millet were present. While further archaeobotanical investigation may clarify nuances in the farming system, it appears that cereal crops dominated cultivation systems across western Xinjiang during the period from ca. 2000–1200 BC.

The dominance of cereals likely reflects long-standing traditions in farming systems across the mountains and deserts of Central Asia, where these cultivation strategies likely originated^46^. Many scholars have noted similarities in cultural aspects and cultivation technology among peoples in the mountains of Central Asia and those in western Xinjiang during this period^7^. In this regard, the dispersal of early farming traditions may have followed, what Frachetti^11^ referred to as the Inner Asian Mountain Corridor (Fig. 6b).

Based on the archaeobotanical, isotopic, and archaeological evidence, we think there is reason to believe that occupation and cultivation of crops at the Wupaer site changed between the early (1500–1300 BC) and late periods (1200–400 BC). Counting naked and hulled forms of barley separately, six grain crops and at least one legume were present in the late Wupaer period (1200–400 BC) (Fig. 4). It is worth noting this is the first identification of a legume crop in an archaeobotanical record from the Taklimakan Desert. Additionally, it is the first attempt to subdivide hulled barley into two- and six-rowed forms. When taken in combination with the greater archaeological visibility of the later period, it seems likely that there was an intensification of farming strategies after 1200 BC. The greater archaeological visibility likely correlates with a greater population, which may have fed into the increased exchange at this period.

Similar shifts in cultivation practices have been observed in other areas of Inner Asia, for example, in northern Xinjiang, naked barley was replaced by varieties of hulled barley and legumes^47^. Spengler^45^ presented the possibility that the shifts in dominant barley varieties across Central Asia during the first millennium BC may be tied into changes in investment in irrigation. Two- and six-rowed hulled barley was widely cultivated across southern Central Asia before the crops were introduced into Xinjiang. For example, two-rowed specimens have been recovered, dating as early as 5000 BC at Chagylli and Togolok in Turkmenistan^48^, and six-row barley grains were found at Aunu (4500–1700 BC)^47^, Gonur (Phase I, 2400–1950 BC)^49^, and Sarazm (ca. 3500–2000 BC)^50^. Preserved ancient peas have been found across a wider region, including Anau South (3000–1700 BC)^49^, Adji Kui 1 (2400–1300 BC)^51^, 1211/1219 (FS20, 1400 BC)^41^, and possibly Gonur (Phase I, 2400–1950 BC)^49^ in southern Turkmenistan, as well as Tasbas (2a, 1441–1262 BC)^52^ in the far east of Kazakhstan. As the core region of the Oxus cultural milieu, peoples in southern Central Asia had developed a prosperous agricultural tradition. Wheat, barley, and legumes had been widely utilized by agriculturists across the mountains and deserts of Central Asia before the second millennium BC^46,49,52^. Compared with developed irrigation agriculture in southern Central Asia, agriculture in northern Central Asia was usually associated with low-investment crops, such as naked barley and millets, which were characterized by wider tolerance in water and temperature but lower yields^44,52,53^.

The proportion of naked barley grains recovered from sites outside Xinjiang, notably in southwest Asia and Europe declined and gradually disappeared during the first millennium BC^47^. While our data are not robust enough to make an exact analogy, it is interesting to note that, at the Wupaer site, naked barley seems to disappear after 1200 BC. This may reflect a choice in cultivation by famers, as naked barley is easy to process and cultivate, but its yield is relatively low, and it is more suitable for small-scale population cultivation and processing. After the emergence of efficient processing tools such as millstones and irrigation systems in the first millennium BC, hulled barley with higher yields became more polular^47^.

Peas have been reported at the site of Qasim Bagh (2000–1500 BC) and Kanispur (2700–2000 BC) in the Kashmir Valley of the Pamir Mountains^43,54^. *Vigna* sp. became a common crop in South Asia at least five millennia ago^55,56^, and entered eastern China around 2000 years ago^57^. Interestingly, mountain passes, such as the Swat and Kashmir Valleys historically served as routes of connection between the northern Indus and Central Asia or Xinjiang. Therefore, it is likely that new crops were traded through these valleys during the late Wupaer period (1200–400 BC), and would have been introduced into Kashgar Oasis by agriculturists from southern Central Asia (Fig. 6b).

Agricultural populations did not only introduce new crops, but they reshaped the structure of agriculture across the Tarim Basin. Wheat was clearly a prominent part of the economy by the tail end of the second millennium BC, and may have become more important than barley at Wupaer (Fig. 6a). At other sites in the Taklimakan Desert, with wheat, such as Qunbake Cemetery (955–680 BC) of Luntai County^58^, iron sickles were also found^59^. Wheat grains were also found at the ancient urban center of Yuansha (Djoumboulak Koum) (ca. 400–0 BC)^23,24^ and at the Sampula Cemetery (mid-first millennium BC)^60^.

### Water management in the Tarim Basin

Wheat is one of the most important cultivated crops in the world^61^, but it has a long cultivation period and it is water demanding, which made it difficult to intensify before mechanized labor. Stable carbon isotope (δ^13^C) analyses of ancient grains can help interpret the level of water supply available to those crops, and therefore, provides a rough estimate for assessing grain yields. Researchers have suggested that water input during the growing period is positively correlated with a carbon isotope discrimination (Δ^13^C) value of wheat grains^62,63^.

**Figure 5:**
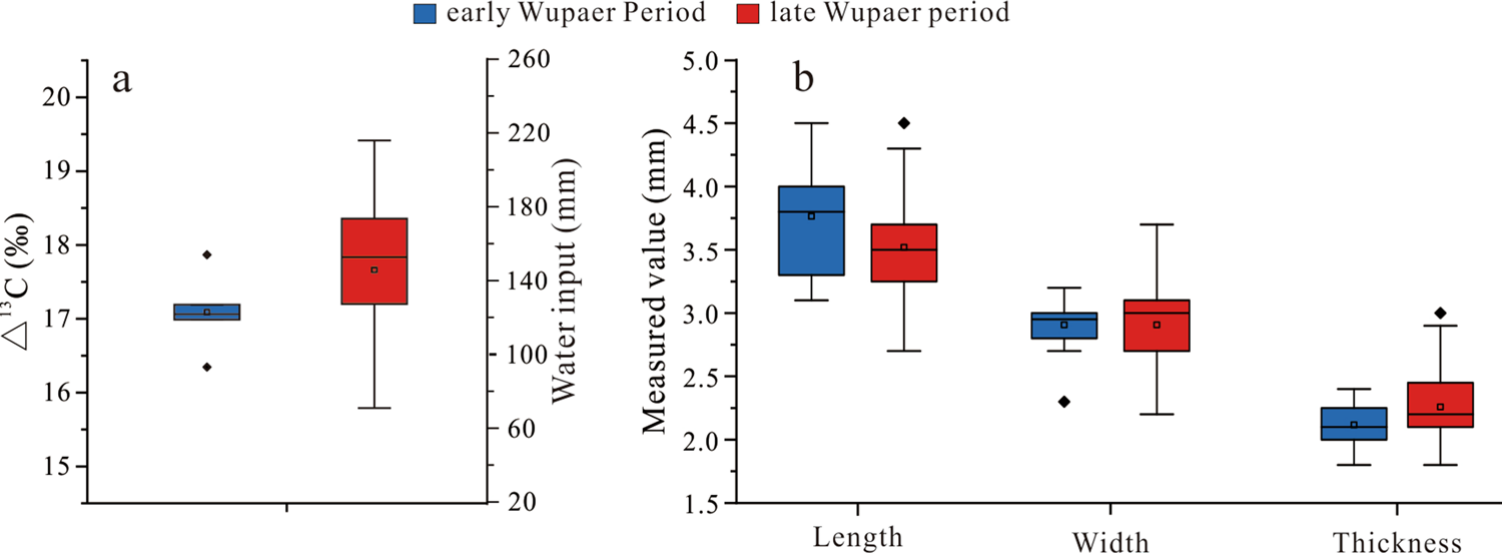
(**a**) The Δ^13^C value and water input of wheat at Wupaer, (**b**) the morphology parameter of compact wheat at Wupaer.

The boxplot of Δ^13^C values and reconstructed water input of ancient grains from Wupaer shows that wheat during late occupation period generally had higher Δ^13^C values than the early period (Fig. 5a and Table S2). Paleoclimatic studies in this part of the world do not suggest that a shift to more humid conditions occurred in the regions during the transitional period^64^. Therefore, we suggest that the best explanation for the differences in water input was provided to wheat by cultivators during its growth period. Obviously, there are many factors that can affect water input and carbon isotope levels, but given that a greater management of water through gravity irrigation of mountain melt streams is expected at this period, the isotope data seems to support the archaeology^65–67^.

Paleoecologists have proposed that as early as the second millennium BC, salinization of top soils, possibly caused by irrigation, was already occurring at the Xintala site^22^. The irrigation systems found in Mohuchahangoukou^67^ and at the Yuansha ancient city (Djoumboulak Koum) (ca. 400–0 BC)^23,24^ suggest that people were significantly improving water management technology. It is reasonable to assume, given the isotope values, that farmers in the Wupaer region might have irrigated wheat, despite the fact that archaeologists were unable to identify ancient canals on the heavily wind-deflated landscape.

Improved ability for water management is a sign of increased productivity and time investment in sedentary economic practices, but the size and shape of wheat grains do not appear to be affected by the increased water input. The measurements of wheat morphology show that compact wheat grain size during the late Wupaer period was not larger than that during the early Wupaer period (Fig. 5b). This would support assumptions that the highly compact morphotypes, as discussed by Spengler^45^, reflect non-plastic genetic mutations or aspects of complex spherococcoid syndromes. In some parts of Asia, dwarfing of semi-dwarfing traits in cereals are linked to ecological adaptations, such as greater drought tolerance, higher yields, and tolerance to heavy snow cover^45,46^. The highly compact grains in the Taklimakan Desert after 1200 BC, may suggest that semi-dwarfing varieties of wheat were preferred given their adaptability to the extreme continental climate of the Tarim Basin.

### The emergence of city-states in the Taklimakan Desert

One of the most heavily debated topics in Central Asian archaeology is how best to model changes in social orders over time, especially during a period often referred to at the Bronze/Iron Age Transition (ca. 1200–700 BC). Traditional models of social development in Central Asia claim that this period marked the first appearance of highly specialized mobile pastoralists^30,68–70^. The heavy focus on socio-political dynamics is tied directly into the assumption that all people in this broad part of the world lacked agriculture, making them a unique case study for imperial formation in the absence of grain surplus^71–74^. Scholarship in Central Eurasia often focuses on linear complexity models^11,72^, claiming that social orders in Central Asia were different in prehistory than in parts of the world where agricultural surplus was tied into sedentism and demographic expansion. While it is beyond the scope of this paper to discuss the specific nature of political systems in the oases of Xinjiang, they are often likened to city-states or small-scale polities. Archaeological evidence from across Xinjiang illustrate a greater density of sites and more expansive settlements during the first millennium BC^7^. Understanding what role irrigation played in these cultural changes in this hyper arid region will provide a reference for discussing the Bronze/Iron Age Transition in the arable mountain valleys across Eurasia.

At the period of this supposed switching to more mobile and specialized pastoral economies, archaeobotanical data have illustrated that an intensification of agriculture was underway and more dense farming villages were forming in the mountain foothills to the west^75^. Chang^76^ has argued for a much more intertwined economic system across the Tien Shan Mountains during the first millennium BC, and Spengler et al.^77^ have suggested that the intensification of farming through irrigation was a key driver of socio-political changes in Central Asia. Li^7^ recently discussed the intensification of irrigated farming in northern regions of Xinjiang, and other scholars have discussed the increasingly more prominent role of irrigation in farming in the foothills of Central Asia^78^. Miller et al.^79^ suggest that even more intensive crop-rotation systems were being implemented in parts of Central Asia by the mid-first millennium B.C. and Spengler et al.^80^ have discussed the importance of these complex multi-cropping systems in the Pamir Mountains by the tail end of the second millennium BC. Archaeobotanical data have also been used to demonstrate an increased prevalence of water-demanding crops in southern Central Asia^79^, and more intensive irrigation systems may have existed from the Murghab Oasis^81^, to Khorezm^82^, and Semirechye^77^ in the late first millennium BC. Wilkin et al.^83^ recently suggested that the introduction of millet and possibly cereal farming in Mongolia may have played a role in the development of more complex social systems there as well. The apparent switch to more heavily irrigated crops during the key transitional period in the oases on the peripheries of the Taklimakan Desert, further support the growing evidence that illustrate a link between more elaborate and hierarchical political systems and farming in Central Asia^43^.

**Figure 6:**
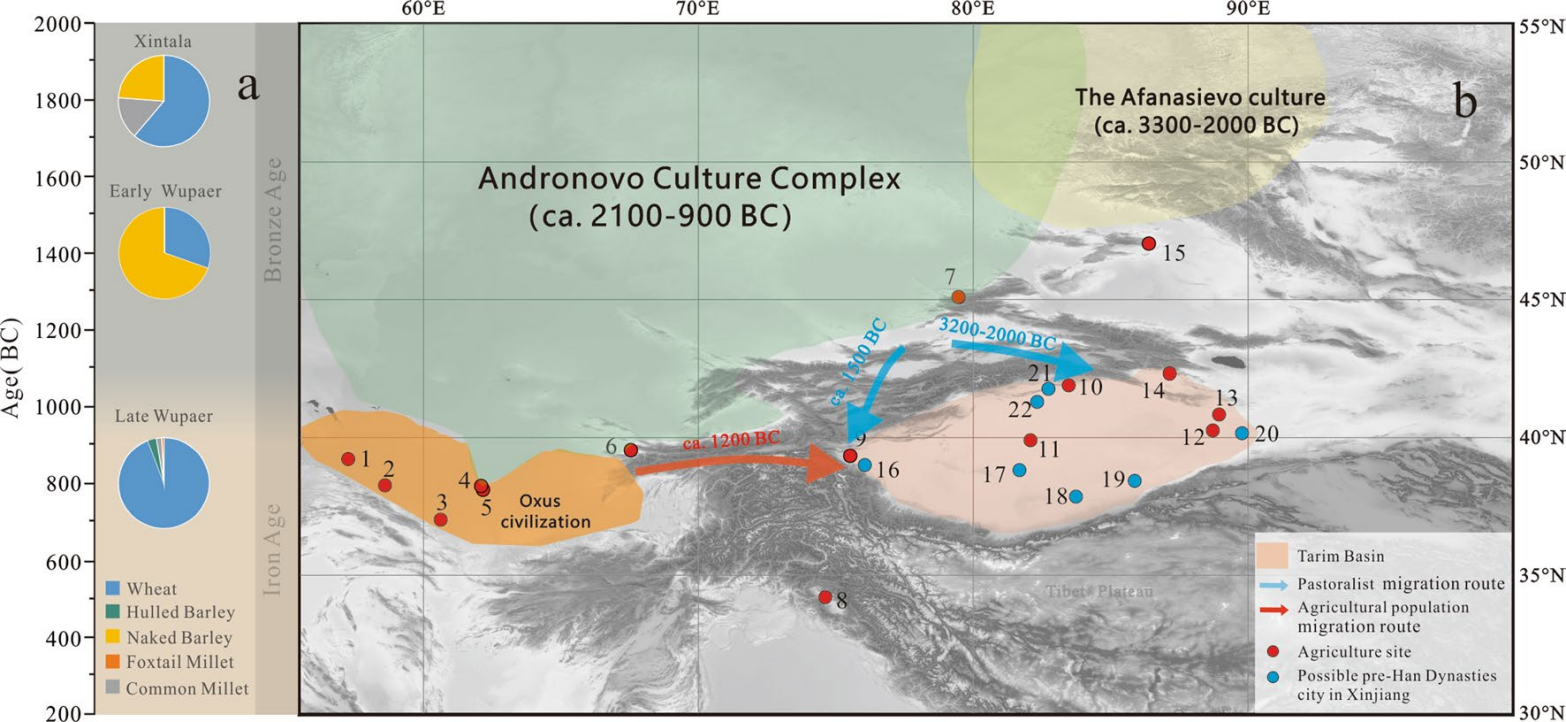
(**a**) The crop structure succession reveals agricultural shifts in the oases of the Taklimakan Desert began during the very end of the second and early first millennia BC. (**b**) Map shows proposed routes of crop dispersals and technology spread into the Tarim Basin (DEM date derives from Geospatial Data Cloud https://www.gscloud.cn and the DEM date is edited by Global mapper). (1) Togolok (ca. 5000 BC), (2) Anau (4500–1700 BC), (3) Chagylli (ca. 5000 BC), (4) Gonur (Phase I, 2400–1950 BC), (5) 1211/1219 (ca. 1950–1300 BC), (6) Sarazm (ca. 3500–2000 BC), (7) Tasbas (1441–1262 BC), (8) Qasim Bagh (4000–3500 BP), (9) Wupaer (1500–1300 BC and 1200–400 BC), (10) Qunbake (955–680 BC), (11) North Keriya (close to Xiaohe Cemetery), (12) Xiaohe (1691–1292 BC), (13) Gumugou (1886–1746 BC), (14) Xintala (1920–1530 BC), (15) Tongtian Cave, (16) Ahetu, (17)Yuansha (ca. 400–0 BC), (18) Andier, (19) Qiemo, (20) Loulan (ca. 200 BC–400 AD), (21) Qiuci, (22) Wushikate.

Scholars have suggested that increasing the labor input into irrigation might have accelerated the emergence of city states^9^. According to historical texts^2,3,84^, more than 25 city-states were present in this region when the envoy of Zhang Qian arrived. He was appointed as ambassador by the emperor of the Han Dynasty to contact counties to the west of the imperial boundaries and reached this region in 129 BC. Considerable ruins of ancient towns of proto-urban centers dating before the Han Dynasty (before 202 BC) exist across the Taklimakan, such as Ahetu, Yuansha (Djoumboulak Koum), Andier, Qiemo, Loulan, Qiuci, and Wushikate^1^ (Fig. 6b). Some of these ancient city ruins have been directly dated; for example, radiocarbon dating results show that Yuansha (Djoumboulak Koum) was constructed between ca. 400–0 BC (recalibrated by inCal 13, 2σ)^24,29,85^. Likewise, human activities at Loulan ancient city began as early as 200 BC^86^. Historical and archaeological data indicate that the oasis cities of the Taklimakan Desert were constructed before the colonization of the region by the West Han Dynasty^9^.

Supporting the discussions of crop dispersal discussed above, mtDNA analyses of fifteen human remains excavated from Yuansha (Djoumboulak Koum) suggests a relatively close relationships with modern populations of southern Central Asia and the Indus Valley, as well the ancient population of Chawuhu^23,24,85^. This conclusion further indicates bilateral connections between oasis populations in southern Central Asia and arid western China (Fig. 6b). Scholars have already suggested that the irrigation technology present in Xinjiang in prehistory may have spread into the region through the mountains of Central Asia from southern Central Asia^67^.

The multiproxy data that we present in this study leads us to conclude that early cities in the Tarim Basin were closely related to the populations from southern Central Asia. Eastward-moving agriculturists from southern Central Asia introduced the new crops, including legumes, and new irrigation technology into the Tarim Basin after 1200 BC (Fig. 6b). All of these factors likely articulated into a complex cultural realm leading to increased populations, the formation of large oasis towns, and increased exchange along the proto-Silk Road.

## Methods

### Radiocarbon dating

Seven carbonized seeds from three pits were chosen for dating by Accelerator Mass Spectrometry (AMS). The pretreatment process included: (1) cleaning the surface of the samples; (2) treating the samples with an acid–alkali-acid treatment; (3) combusting the sample to produce carbon dioxide; and (4) reducing it to graphite. Radiocarbon ages were measured at the Australian Nuclear Science Technology Organization (ANSTO) in Sydney, Australia. Other dates were measured at Beta Analytic in Miami, USA, which served as comparison between the two labs. The radiocarbon ages were calibrated by using the IntCal13 calibration curve in Oxcal v4.3.2^87^.

### Archaeobotanical analyses

The cluster of sites at Wupaer is highly wind deflated and most sites consisted of surface scatters, as is characteristic of archaeological sites in arid regions of Eurasia. The lack of stratigraphic integrity hinders archaeobotanical investigation, but after a close survey of the sites we identified a few cultural layers and ash pits with secure contexts. We collected 11 sediment samples of 20-L at three of the sites in the cluster, the sites were designated as Wupaer 1, 2, and 3 (5 samples, 3 samples, and 3 samples were collected respectively). Flotation of the sediments was performed at a nearby fresh-water spring, using a basic bucket method, and screens of 0.3 and 0.6 mm.

The samples were placed in a bucket filled with water and manually agitated with a clean wooden stick. Secondly, the floating carbonized remains were decanted into a screen and were cleaned in spring water. Lastly, carbonized remains were collected in cloth bags and dried in the shade. All carbonized remains were identified and measured under a stereoscopic microscope (Leica M205c) in the laboratory of Institute of Vertebrate Paleontology and Paleoanthropology (IVPP), Chinese Academic of Science (CAS), Beijing. Seed identification in this study relied on published archaeological and modern data^88,89^ as well as the modern plant seed bank of the environmental archaeology laboratory of IVPP.

### Carbon isotope analysis

Fifteen carbonized wheat grains were selected for measuring the ratio of organic carbon isotopes. The pretreatment process proceeded as follows: (1) carbonized seeds were ground into powder by using an agate mortar and seed powder was placed into a small beaker; (2) carbonate was removed by hydrochloric acid then washed to neutrality by using distilled water; (3) roughly 15 mg of dried powder was placed into a silver box and compressed into a small ball with tweezers; and (4) the sample was placed into the chamber for measuring the ratios of organic carbon isotopes. All samples were measured by using a MAT 253 Mass Spectrometer in the isotope laboratory of the IVPP, Chinese Academic of Science, Beijing.

In order to assess the available water supply for growing wheat plants during seed formation, equations proposed by Farquhar et al.^90^ and Araus et al.^91^ were used, as follow:

1 $$\Delta^{13} {\text{C}}=\frac{{\delta_{{\text{a}}} - \delta_{{\text{p}}} }}{{1 + \delta_{{\text{p}}} }}$$

where Δ^13^C is carbon isotope discrimination, δ_a_ and δ_p_ are δ^13^C of the atmosphere and the δ^13^C of wheat grains. The δ_a_ date has been obtained from air bubbles trapped in Antarctic ice cores^92^.

2 $${\text{WI}} = 39.94 \times \Delta^{13} {\text{C }} - 560.36$$

where WI is water input, Δ^13^C is carbon isotope discrimination.

## Supplementary information

Supplementary Tables.
