# Supplementary material for: Prehistoric agriculture and social structure in the southwestern Tarim Basin: multiproxy analyses at Wupaer
Source: Sci Rep. 2020 Aug 28;10:14235. doi: 10.1038/s41598-020-70515-y (PMC7455698; doi:10.1038/s41598-020-70515-y)
Supplement: Supplementary file 1 — Supplementary Tables. [file 41598_2020_70515_MOESM1_ESM.docx]

**Prehistoric Agriculture and Social Structure in the Southwestern Tarim Basin: Multiproxy analyses at Wupaer**

Qingjiang Yang ^1,2,3^, Xinying Zhou ^1,2,3*^, Robert Nicholas Spengler ^4^, Keliang Zhao ^1,2,3^, Junchi Liu ^1,2,3^, Yige Bao ^1^, Peter Weiming Jia ^5^, and Xiaoqiang Li ^1,2,3^

1. State Key Laboratory of Vertebrate Evolution and Human Origin, Institute of Vertebrate Paleontology and Paleoanthropology, Chinese Academy of Sciences, Beijing,100044, China

2. CAS Center for Excellence in Life and Paleoenvironment, Beijing, 100044, China

3. University of Chinese Academy of Sciences, Beijing,100049, China

4. Max Planck Institute for the Science of Human History, Jena, 07745, Germany

5. Department of Archaeology, The University of Sydney, Sydney, NSW 2006, Australia

| Site | Sample code | Compact wheat | Common wheat | Two-row barley | Six-row barley | Naked barley | Common millet | Foxtail millet | Pea | *Vigna* sp. | Camelthorn | Cockleburs | Lesser grain borers |
| --- | --- | --- | --- | --- | --- | --- | --- | --- | --- | --- | --- | --- | --- |
| Wupaer 1 | 1 | 3 | 0 | 0 | 0 | 0 | 0 | 0 | 1 | 0 | 0 | 0 | 0 |
|  | 1-2 | 21 | 0 | 1 | 0 | 0 | 7 | 3 | 1 | 0 | 0 | 0 | 0 |
|  | 2 | 5 | 0 | 0 | 0 | 0 | 2 | 0 | 0 | 0 | 1 | 0 | 0 |
|  | 3 | 3 | 0 | 0 | 0 | 0 | 0 | 0 | 0 | 0 | 0 | 0 | 0 |
|  | 4 | 1 | 0 | 0 | 0 | 0 | 0 | 0 | 0 | 0 | 0 | 0 | 0 |
|  | Subtotal | 33 | 0 | 1 | 0 | 0 | 9 | 3 | 2 | 0 | 1 | 0 | 0 |
| Wupaer 2 | 1 | 67 | 6 | 2 | 6 | 0 | 0 | 0 | 1 | 2 | 3 | 3 | 0 |
|  | 2 | 140 | 10 | 0 | 2 | 0 | 2 | 0 | 0 | 0 | 2 | 0 | 0 |
|  | 3 | 271 | 0 | 0 | 6 | 0 | 0 | 1 | 0 | 0 | 1 | 0 | 2 |
|  | Subtotal | 478 | 16 | 2 | 14 | 0 | 2 | 1 | 1 | 2 | 6 | 3 | 2 |
| Wupaer 3 | 1 | 1 | 0 | 0 | 0 | 13 | 0 | 0 | 0 | 0 | 0 | 0 | 0 |
|  | 2 | 0 | 0 | 0 | 0 | 20 | 0 | 0 | 0 | 0 | 0 | 0 | 0 |
|  | 3 | 20 | 0 | 0 | 0 | 15 | 0 | 0 | 0 | 0 | 0 | 0 | 0 |
|  | Subtotal | 21 | 0 | 0 | 0 | 48 | 0 | 0 | 0 | 0 | 0 | 0 | 0 |
|  | Total | 532 | 16 | 3 | 14 | 48 | 11 | 4 | 3 | 2 | 7 | 3 | 2 |

**Table S1.** The statistical result of carbonized remains at the Wupaer site

| δ^13^C (‰) | | △^13^C (‰) | | Water input (WI) (mm) | |
| --- | --- | --- | --- | --- | --- |
| Early Wupaer  (1500-1300 BC) | Late Wupaer  (1200-400 BC) | Early Wupaer  (1500-1300 BC) | Late Wupaer  (1200-400 BC) | Early Wupaer  (1500-1300 BC) | Late Wupaer  (1200-400 BC) |
| -22.9 | -23.1 | 17.0 | 17.2 | 118 | 126 |
| -22.3 | -25.3 | 16.3 | 19.4 | 92 | 215 |
| -23.1 | -22.1 | 17.2 | 16.1 | 126 | 83 |
| -23.0 | -24.0 | 17.1 | 18.0 | 121 | 160 |
| -23.8 | -23.3 | 17.9 | 17.3 | 153 | 132 |
|  | -24.1 |  | 18.2 |  | 165 |
|  | -21.8 |  | 15.8 |  | 71 |
|  | -23.6 |  | 17.6 |  | 144 |
|  | -24.5 |  | 18.6 |  | 182 |
|  | -24.3 |  | 18.3 |  | 172 |

**Table S2.** The δ^13^C and △^13^C value of 15 carbonized compact wheat and the constructed water input values at the Wupaer site

|  | Length  range | Average  length | Width  range | Average  width | Thickness  Range | Average  thickness | statistical  magnitude |
| --- | --- | --- | --- | --- | --- | --- | --- |
| Compact wheat | 2.4-4.5 | 3.46 | 1.6-3.7 | 2.8 | 1.5-3 | 2.19 | 67 |
| Common wheat | 2.9-4.8 | 3.87 | 1.8-2.9 | 2.26 | 1.5-2 | 1.8 | 14 |
| Naked barley | 3.5-5.5 | 4.49 | 2.5-3.8 | 3.21 | 1.8-2.8 | 2.26 | 18 |
| Two-row barley | 6 |  | 3.3 |  | 2.7 |  | 1 |
| Six-row barley | 3.5-4.5 | 4.22 | 1.9-2.7 | 2.32 | 1.4-2 | 1.82 | 6 |
| Common millet | 1.6-1.8 | 1.7 | 1.5 | 1.5 | 1-1.1 | 1.05 | 3 |
| Foxtail millet | 1.8 |  | 1.3 |  | 1.2 |  | 1 |
| *Vigna* sp. | 3.1-3.2 | 3.15 | 2.2-2.3 | 2.25 | 2-2.1 | 2.05 | 2 |
| Pea | 3 |  | 2.9 |  | 2 |  | 1 |

**Table S3.** The parameters of carbonized crops and legumes at the Wupaer site.
